# Decoding inflammation to target hybrid diabetes phenotypes

**DOI:** 10.3389/fendo.2026.1922969

**Published:** 2026-10-15

**Authors:** Giada Rossi, Annalisa Terranegra, Maria Elena Lunati, Ida Pastore, Lorenzo Pozzi Canova, Loredana Bucciarelli, Vincenzo Cimino, Cristian Loretelli, Moufida Ben Nasr, Paolo Fiorina, Francesca D’Addio

**Affiliations:** 1 International Center for T1D, Pediatric Clinical Research Center Romeo ed Enrica Invernizzi, Department of Biomedical and Clinical Sciences, Università di Milano, Milan, Italy; 2 Translational Medicine Department, Sidra Medicine, Doha, Qatar; 3 Division of Endocrinology, Azienda Socio-Sanitaria Territoriale (ASST) Fatebenefratelli-Sacco, Milan, Italy; 4 Department of Biomedical and Clinical Sciences, Università di Milano and Pio Albergo Trivulzio Hospital, Milan, Italy; 5 Nephrology Division, Boston Children’s Hospital and Transplantation Research Center, Brigham and Women’s Hospital, Harvard Medical School, Boston, MA, United States

**Keywords:** double diabetes, inflammation, obesity, omics, type 1 diabetes

## Abstract

With the escalating global epidemic of overweight and obesity worldwide, metabolic abnormalities (i.e., central obesity, insulin resistance, dyslipidemia and hypertension) are raising in patients with type 1 diabetes (T1D). On the other hand, data are emerging on the detection of autoimmune markers in obese individuals previously diagnosed with type 2 diabetes (T2D). These overlapping immune-metabolic features, characterizing hybrid phenotypes of diabetes such as double diabetes (DD) and latent autoimmune diabetes in adults (LADA), add a new layer of complexity in the diabetes spectrum and increase the risk of diabetes-related complications and mortality. Low-grade chronic inflammation is thought to play a pivotal role in the pathogenesis of DD and LADA and may represent a major driver in β-cell dysfunction and metabolic dysregulation. However, the literature on the topic is still limited, as well as the availability of specific therapeutic clinical approaches. This review aims to summarize current evidence on inflammatory and immune-metabolic biomarkers for early detection and identification of DD and LADA as compared to other types of diabetes. Novel diagnostic strategies, including genomics, miRNomics, metabolomics and microbiomics analysis, together with therapeutic options targeting metabolic and inflammatory pathways will be also explored. Challenges, including disease heterogeneity will be discussed alongside future directions, emphasizing the need for personalized medicine and longitudinal studies to shed more light on this overlooked hybrid phenotypes of diabetes.

## T1D and obesity

### Obesity in T1D

The prevalence of obesity in type 1 diabetes (T1D) is growing worldwide. The DCC/EDIC study showed an increase in obesity from 2% at baseline (1983-1989) to 28% at 12 years of follow-up in patients with T1D (1). Similarly, the SWEET international registry reported overweight or obesity in 31.8% of children and adolescents with T1D (2). The mechanisms behind obesity and insulin resistance in T1D include genetics, epigenetics, enteroendocrine hormones, microbiome, and environmental factors, with effects on multiple organs (3, 4). However, the pathophysiology of energy balance in T1D is still not fully defined. Despite several mechanisms may be responsible for weight gain in individuals with T1D, exogenous insulin treatment is considered one of the major contributors. Indeed, intensive insulin therapy in individuals with T1D has been associated with reduction in long-term risk of complications but, more recently, with weight gain and insulin resistance (5, 6). In line with this, several mechanisms whereby insulin-related weight gain may develop in T1D have been proposed. First, the administration of exogenous insulin in treatment of T1D may not fully mimic physiological insulin action, as subcutaneous delivery of insulin analogues bypasses liver metabolism, resulting in peripheral hyperinsulinemia and hepatic hypoinsulinemia. Chronically elevated levels of insulin in peripheral tissues may increase oxidative stress and inflammation, and modulate expression and activity of insulin receptors, thus worsening the degree of insulin resistance, hyperglycemia and fat accumulation (4). Second, as people achieve an improved glycemic control with insulin therapy, blood glucose concentrations fall below the renal threshold, thereby increasing the conservation of ingested calories (4). Third, alterations in pathways involved in maintaining body composition by balancing anabolism and catabolism, like the growth hormone or IGF-1 system, have been associated to exogenous insulin administration (4). Repetitive episodes of hypoglycemia induced by insulin therapy or the presence of the so called ‘fear of hypoglycemia’, together with emotional eating disorders that may affect T1D patients, can also result in maladaptive eating habits which promote weight gain. Additionally, recent cultural, social and lifestyle changes that favor unhealthy diets and sedentary lifestyles, likely contribute to overweight and obesity in T1D. Finally, genetic and phenotypical factors (i.e., variants of the fat mass and obesity-associated gene, family history of type 2 diabetes, age, diabetes duration, etc.) may further predispose individuals with T1D to weight gain (4, 6–8). Taken together, weight gain associated with intensive insulin therapy increases insulin resistance, leading to higher insulin dose requirements, which in turn promotes further insulin-associated weight gain in a vicious cycle that sustains the tight relationship between autoimmune diabetes and obesity (**Figure 1**).

**Figure 1:**
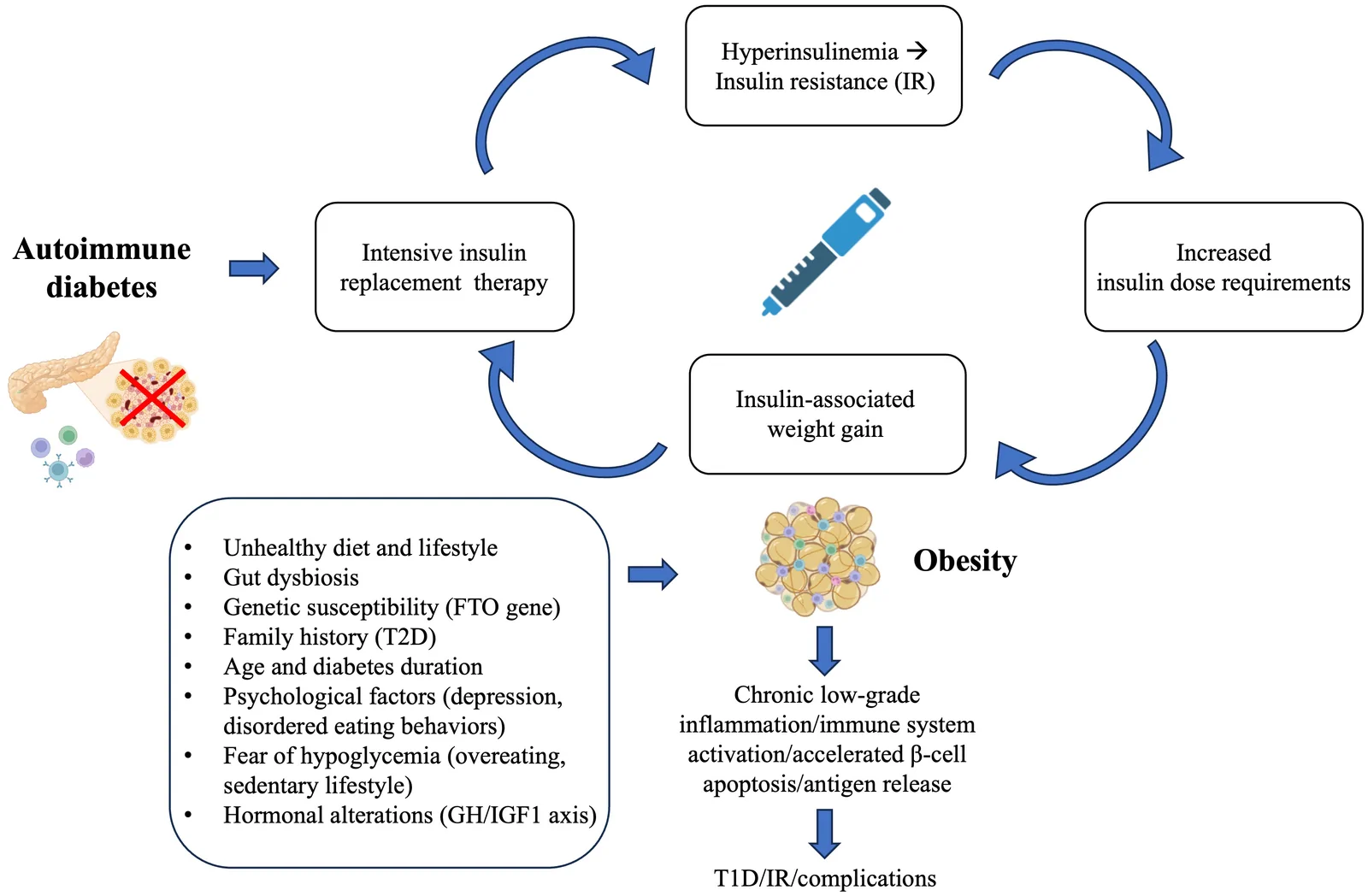
The bidirectional interplay between autoimmune diabetes and obesity. FTO, fat mass and obesity-associated gene; T2D, type 2 diabetes; GH, growth hormone; IGF1, insulin-like growth factor; T1D, type 1 diabetes.

### Autoimmune markers in obese patients

Observational cohort studies on a total of 1.7 million individuals reported a pooled risk ratio of 1.87 (95% CI 1.52-2.29) for T1D among people with overweight or obesity, which reached a risk ratio of 2.55 (95% CI 1.11-5.86) for T1D verified with islet autoantibodies data (9). Moreover, overweight and obesity have been associated with an accelerated development of islet autoimmunity and T1D onset in children and adolescents (10, 11). Several mechanisms may be considered to explain the link between obesity and the increased incidence of T1D. The accelerator hypothesis focuses on obesity-driven insulin resistance in at-risk individuals, which leads to glucotoxicity and advances pancreatic β-cells apoptosis, thereby increasing β-cells-related immunogenicity and further promoting the onset of T1D (12). Similarly, according to the overload hypothesis, excessive body weight in individuals with triggered autoimmunity results in insulin resistance, which induces β-cells to overwork and fasten their apoptosis (13). Moreover, the adipose tissue itself has been shown to impact the function of the immune system. Chronic systemic inflammation related to obesity plays a critical role in the deregulation of T-helper (Th) 17/T regulatory cells (Treg) balance and increases the number of Th1 cells (14). During obesity, a low number of B-regulatory and invariant Natural Killer cells is detectable in adipose tissue, which may favor the infiltration of inflammatory cells, a decreased insulin sensitivity, and a dysregulated adipokines secretion (15, 16). The increase in lipolysis mainly due to local inflammation, hormonal disturbances, and insulin resistance stimulates the release of free fatty acids which in turn promotes pro-inflammatory M1-macrophages infiltration and further activates NOD-like receptor protein 3 (NLRP3)-inflammasome. This lastly leads to increased secretion of interleukins, particularly IL-1β and IL-18 involved in the pathogenesis of autoimmune diseases (17, 18). Moreover, adipocytes may also undergo apoptosis and release antigens that were previously invisible to the immune system, thereby inducing an autoreactive immune response (19, 20). Another hypothesis indicates that a diet with high fat, salt, and simple carbohydrate content could induce metabolic stress that may increase β-cells frailty and susceptibility to develop T1D (21). Ultimately, dysbiosis related to high fat, simple carbohydrates and low fiber diets may constitute an additional link between diet, obesity and T1D (13). Overall, a bidirectional relationship between obesity and autoimmune diabetes has been described, with T1D predisposing to weight gain, and obesity increasing the risk of autoimmunity (**Figure 1**).

### The hypothesis of double diabetes and risk of complications

The growing body of evidence showing that overweight, obesity and metabolic abnormalities frequently coexist nowadays in individuals with T1D, coupled with the detection of autoimmune markers in obese or insulin-resistant subjects points to a progressive convergence between metabolic and autoimmune disfunctions (3, 22). Within this continuum, double diabetes (DD) and latent autoimmune diabetes in adults (LADA) have been described as two hybrid conditions. DD refers to patients with T1D who are overweight, with a family history of type 2 diabetes (T2D) and/or exhibiting clinical features of insulin resistance and metabolic abnormalities typical of T2D (23, 24). LADA represents a slowly progressive autoimmune diabetes occurring in adults who often display clinical and metabolic characteristics that parallel T2D at onset (25). While DD and LADA exhibit overlapping metabolic and immunological features of T1D and T2D, they represent distinct pathophysiological phenotypes arising from varying contributions of β-cell autoimmunity, insulin resistance and metabolic inflammation. In particular, DD has been associated with a markedly higher incidence of micro- and macro-vascular complications, even after adjustments for age, sex, diabetes duration and glycemic control (24). In addition to an increased rate of complications, mortality is also augmented in individuals with DD. The hazard ratio for diabetes-related mortality from the FinnDiane study was significantly higher in individuals with DD as compared to those with T1D but without metabolic syndrome, with an adjusted hazard ratio of 2.52 (95% CI 1.53-4.16) (1). A key element of DD that may increase the risk of developing chronic complications is, indeed, the insulin resistance and the subsequent need for higher doses of subcutaneous exogenous insulin administration. Alterations in traditional cardiovascular risk factors, such as dyslipidemia and hypertension, are likely to play a role in the elevated rate of complications as well. Moreover, insulin resistance and obesity concur to generate a low-grade inflammatory milieu, which aggravates both insulin resistance and atherogenic LDL cholesterol oxidation, thus promoting endothelial dysfunction and vascular occlusion (24, 26). Recognizing these hybrid phenotypes and elucidating their underlying mechanisms is therefore crucial to develop individualized prevention and treatment strategies with the aim of reducing the long-term burden of the disease-related complications.

## Genetic and multi-omics insights into autoimmune diabetes heterogeneity

Precision medicine in autoimmune diabetes seeks to move beyond age-based and phenotype-based classification by integrating genetic susceptibility, early-life exposures, and multi-omics biomarkers to define biologically meaningful disease subtypes. In this framework, the integration of clinical phenotyping with genetic, transcriptomic, metabolic, and microbiome biomarkers collectively inform risk prediction, differential diagnosis, and prognosis (27). Applied to autoimmune diabetes, this integrative strategy reveals how early-life adiposity, immune-metabolic genetic architecture, and dynamic molecular signatures converge to shape autoimmune risk, disease heterogeneity, and clinical trajectories, thereby enabling more precise stratification and the foundation for targeted preventive and therapeutic interventions. Of note, the available molecular and multi-omics evidence is currently more extensive in LADA than in DD, underlying the need for further studies specifically addressing DD.

### Genetics

Mendelian randomization analyses provide robust evidence supporting a causal role of increased childhood adiposity in elevating DD risk, regardless of body size at birth or in adulthood, thereby implicating early-life metabolic load as a driver of autoimmune diabetes susceptibility (28, 29). With regard to genes linked to metabolic function, alterations in the Krüppel-like factor 14 (KLF14) locus may modulate the disease phenotype, with the rs4731702 T allele conferring a more favorable cardiometabolic and inflammatory profile in young individuals with T1D, including improved lipid profile, lower glycated hemoglobin, reduced proinflammatory markers, and increased regulatory T cells frequency (30). Together, these findings underscore how early-life adiposity, epigenetic programming, and genetic modifiers converge to shape DD risk, immune dysregulation, and metabolic outcomes, offering important insights for risk stratification and preventive strategies. The available genetic evidence in LADA is substantially more extensive than DD and revealed a clear distinction of LADA from classic diabetes subtypes, with mixed features. The first GWAS of LADA identified genetic correlations with both T1D and T2D, uncovered a novel signal near the gene encoding 6-phosphofructo-2-kinase/fructose-2,6-biphosphatase 3 (*PFKFB3*), and confirmed attenuation of key T1D-associated HLA haplotypes, underscoring the mixed immune-metabolic architecture of LADA (31). Large-scale genetic analyses demonstrate that LADA shares substantial etiological overlap with T1D, with strong associations at multiple T1D loci, including the major histocompatibility complex region, as well as at gene such as protein tyrosine phosphatase non-receptor type 22 (PTPN22), SH2B adaptor protein 3 (SH2B3), and insulin. Only modest contributions derive from T2D susceptibility variants, aside from a notable signal at HNF1 homeobox A (HNF1A), while the T2D risk allele at transcription factor 7 like 2 (TCF7L2) appears to not be enriched in LADA (32). Additional fine-mapping of the MHC region demonstrates that although class II loci strongly influence LADA risk, the independent class I effects characteristic of childhood-onset T1D are absent in LADA, suggesting these signals may genetically discriminate the two autoimmune subtypes (33). Complementary work assessing PTPN22 polymorphisms shows that the risk-associated c.1858T allele observed in T1D does not confer similar risk in LADA, whereas other PTPN22 variants exhibit distinct allele-frequency patterns and correlate with clinical features, supporting a unique genetic signature for LADA (34). Meta-analytic evidence further highlights population-specific associations between LADA and HLA class II haplotypes, with several DQA-DQB and DRB-DQB combinations conferring increased or decreased risk across Chinese, Japanese, and Caucasian cohorts (35). Collectively, these findings delineate LADA as a genetically heterogeneous but predominantly autoimmune entity, shaped by both shared and distinct determinants relative to T1D and T2D.

### miRNomics

Across multiple transcriptomic and biomarker-focused investigations, circulating non-coding RNAs have emerged as promising tools for improving the differential diagnosis and mechanistic understanding of autoimmune diabetes, including LADA. A transcriptomic study in South China reveals an abnormal neutrophil gene signature in newly diagnosed LADA, suggesting innate immune dysregulation as a potential early biomarker for disease onset (36). Plasma-derived exosomal miRNA profiling revealed 75 dysregulated species in LADA, with three candidates—miR-146a-5p, miR-223-3p, and miR-21-5p—demonstrating strong discriminatory performance between LADA and T2D (37). Complementary serum small-RNA sequencing in a large case–control cohort identified distinct signatures separating autoimmune (T1D and LADA) from non-autoimmune diabetes, including differential expression of mature miRNAs, isomiRs, and a markedly downregulated tRNA fragment, although profiles did not distinguish LADA from T1D (38). Circular RNA analysis further demonstrated extensive dysregulation in LADA and T2D compared with healthy controls, implicating pathways related to tumor-necrosis factor (TNF) signaling, FoxO activity, cellular senescence, and synaptic plasticity; six circRNAs emerged as the most discriminative molecular candidates (39). At the cellular level, miR-143-3p was shown to be upregulated in autoimmune diabetes, where it modulates inflammatory cytokine production by directly targeting the transcription factor component FOS like 2 (FOSL2), suggesting an epigenetic mechanism contributing to immune dysregulation (40). Additional analyses of circulating miRNAs demonstrated significant downregulation of miR-21, miR-25, miR-146a, and miR-181a in autoimmune diabetes compared with T2D and healthy individuals, with miR-25 and miR-146a emerging as independent predictors of autoimmune diabetes, including LADA (41). Finally, comprehensive miRNA transcriptome profiling at diagnosis identified multiple immune-related differentially expressed miRNAs in LADA, several of which showed validated discriminatory power relative to T2D (42). Collectively, these studies underscore the potential of circulating small RNAs as mechanistically informative biomarkers for distinguishing diabetes subtypes and elucidating the immunopathogenesis of LADA.

### Metabolomics

Metabolomic analysis of LADA reveals that this diabetes subtype does not exhibit a unique metabolite profile but rather occupies an intermediate metabolic position between T1D and T2D. In a cohort of 150 patients (50 per group), 123 metabolites were quantified, of which 99 differed between diabetes types. Principal component analysis demonstrated that LADA patients overlapping metabolically with T1D progressed more rapidly to insulin therapy compared with those overlapping with T2D. These findings suggest that LADA represents a continuum rather than a metabolically distinct entity, with its clinical progression influenced by proximity to T1D or T2D metabolic characteristics (43).

### Microbiomics

The gut microbiota and microbial metabolites interact with host genetics and dietary habits to modulate metabolic and immune pathways relevant to T1D risk and progression. Microbiome-focused studies revealed disease-specific alterations in beneficial taxa, notably *Bifidobacterium* species, and their correlations with branched-chain amino acids and short-chain fatty acids, thereby delineating integrated microbial–metabolomic signatures that distinguish obesity and T1D from health (44). Experimental models showed that dietary interventions targeting the microbiota, including fermentable fibers such as inulin and bioactive components like extra virgin olive oil, can delay T1D onset, reduce insulitis, and improve metabolic control through SCFA production, IL-22 signaling, and favorable shifts in microbial ecology (45, 46).

Together, these findings highlight the multifactorial nature of hybrid diabetes phenotypes, shaped by early-life adiposity, genetic and epigenetic regulation, and diet–microbiome–immune interactions, with implications for prevention and precision interventions.

## Therapeutic options available

Lifestyle interventions, including high-protein, high-fiber, low-fat diets with optimal carbohydrate intake, as well as regular physical activity, may improve insulin sensitivity and reduce daily insulin requirements in T1D. Importantly, these interventions can also mitigate the chronic low-grade inflammation that characterize DD and LADA, providing an adjunctive treatment alongside pharmacological therapy (47). With the exception of the amylin analogue pramlintide, which has been approved by the FDA as an adjunct to insulin therapy in individuals with either T1D or T2D, insulin still remains the gold-standard approved and established medical treatment for T1D (48). Immunomodulatory therapies targeting the autoimmune processes, like the anti-CD3 teplizumab, have shown efficacy in preserving β-cell function and delaying disease progression in early disease stages of T1D (49, 50). However, no randomized clinical trials have specifically evaluated such therapies in patients with LADA or DD to date. Conversely, glucose-lowering drugs commonly used for T2D treatment, like metformin, sodium-glucose co-transporter type 2 inhibitors (SGLT2i), dipeptidyl peptidase-4 inhibitors (DPP4i) and glucagon-like peptide-1 receptor agonists (GLP1-RAs), have also shown anti-inflammatory properties, improved insulin sensitivity, reduction in body weight, and favorable cardiometabolic effects, suggesting their potential role as adjunctive therapies in autoimmune diabetes with overlapping metabolic features (**Table 1**) (51–54). While adjunctive metformin therapy may improve insulin sensitivity without significantly impacting glycemic control in T1D, SGLT2i have demonstrated increase in time spent in glucose range 70–180 mg/dL (TIR), and reduction in glycated hemoglobin (HbA1c) levels, body mass index and daily dose of insulin when combined with insulin therapy. However, SGLT2i have been linked to increased incidence of diabetic ketoacidosis in autoimmune diabetes limiting their use in this context (55–59). Accumulating evidence suggests immunomodulatory activity of DPP4i. Randomized controlled trials (RCTs) also indicated that DPP-4i are generally safe in autoimmune diabetes and may modestly improve glycemic control by preserving residual β-cell function in early LADA. Nevertheless, their impact on insulin requirements, insulin sensitivity, and long-term disease progression appears minimal and needs further investigation on larger cohorts of patients (60–63). GLP1-RAs represent other promising candidates for the treatment of DD and LADA. The ADJUNCT trials showed that liraglutide administration together with insulin therapy in individuals with T1D reduced HbA1c level, daily dose of insulin and body weight as compared to placebo, although with an increased risk of hypoglycemia and ketosis (64, 65). Similarly, Edwards and colleagues analyzed 76 individuals with T1D 1 year after the initiation of GLP1-RAs treatment and showed reduction in weight, HbA1c level, total daily dose of insulin, and total and LDL cholesterol, with a minimal increase in diabetic ketoacidosis occurrence (66). Moreover, greater reduction in body weight was found with GLP1-RAs compared to SGLT2i treatment (-5.5% vs -1.9% of body weight with GLP1-RAs vs SGLT2i, respectively) (66). Nonetheless, most of the literature on GLP1-RAs therapy in individuals with autoimmune diabetes derives from retrospective or *post-hoc* analyses and the current knowledge about the overall effects of this therapy in T1D is incomplete (67). Pasqua et al. demonstrated improved glycemic control and reduction of body weight in a randomized, double-blind crossover trial comparing once weekly subcutaneous semaglutide versus placebo in individuals with T1D treated with automated insulin delivery (AID) systems, without diabetic ketoacidosis or severe hypoglycemic episodes (68). In addition, the ADJUST-T1D trial showed that semaglutide treatment, compared with AID use alone, significantly increased the achievement of TIR greater than 70%, together with time below range (TBR) less than 4% and body weight reduction of at least 5% in adults with T1D and obesity (69). Moreover, in the ENDIS study, semaglutide vs placebo showed improvement in the endothelial function and reduction in high-sensitivity C-reactive protein (hs-CRP) levels in individuals with T1D on insulin therapy (70). Finally, in a *post-hoc* analysis of the SURPASS clinical trials, the novel once-weekly dual glucose-dependent insulinotropic peptide (GIP)/GLP-1RA, tirzepatide, provided substantial reductions in HbA1c level and body weight in adult glutamic acid decarboxylase antibody (GADA)-positive patients previously diagnosed with T2D. Interestingly, these effects have been associated with increased values of the marker of β-cell function, HOMA2-B, and decreased values of the marker of insulin resistance, HOMA2-IR (71). Indeed, β-cell function, measured by C-peptide level, and insulin resistance, are considered among the most relevant parameters to be taken into account when evaluating the treatment of patients exhibiting features of both T1D and T2D. Experts suggest that subjects with fasting C-peptide levels below 0.3 nmol/l should be treated with sole insulin therapy; patients with values between 0.3 and 0.7 nmol/l should be treated with glucose lowering drugs associated with basal insulin therapy, according to the characteristics of the patient; finally, individuals with c-peptide values above 0.7 nmol/l should be treated with the same algorithm used for T2D patients (72). In line with this, in a *post-hoc* analysis from the AWARD-2, -4, and -5 trials, the GLP1-RA, dulaglutide, was less effective in reducing HbA1c levels in patients with LADA with low fasting c-peptide levels (≤0.25 nmol/l) (73). Similarly, in a multicenter retrospective analysis in patients with LADA, Lunati et al. reported that semaglutide as add-on therapy to insulin significantly improved glycemic control, reduced body weight and insulin requirements, and increased c-peptide levels and TIR, particularly in those with preserved β-cell function (c-peptide >0.7 nmol/l), without major safety concerns (74). However, no differences related to fasting c-peptide values were observed in GADA-positive patients treated with tirzepatide (71). Overall, given their pleiotropic action, with proven effectiveness in reducing body weight, hyperglycemia and cardiovascular risk, along with their anti-inflammatory and β-cell protective properties (75), GLP1-RAs and GIP/GLP-1RAs are among the most promising adjunctive therapeutic options in the management of DD and LADA, although further confirmatory large studies are needed in autoimmune diabetes.

**Table 1:** Potential therapeutic options already available for double diabetes and LADA management.

Drug (class)	Trial (design)	Study Population	Key results	Reference
Metformin (biguanide)	**REMOVAL** (multicenter RCT, double-blind, placebo-controlled)	Adults with long-standing T1D and cardiovascular risk factors	↓ body weight; ↓ LDL-C; no sustained HbA1c improvement; minimal effect on insulin dose	PIMID: 28615149
Dapagliflozin (SGLT2i)	**DEPICT-1/DEPICT-2** (phase 3 multicenter RCTs, double-blind, placebo-controlled)	Adults with T1D and inadequate glycemic control	↓ HbA1c; ↓ body weight; ↓ total daily insulin	PIMID: 33145944
Empagliflozin (SGLT2i)	**EASE-2/EASE-3** (phase 3 multicenter RCTs, double-blind, placebo-controlled)	Adults with T1D	↓ HbA1c; ↓ weight; ↓ insulin dose (dose-dependent effects)	PIMID: 30287422
Empagliflozin (SGLT2i)	Randomized, double-blind, placebo-controlled crossover trials	Adults with T1D on AID/PLGS systems and suboptimal control	↑ TIR	PIMID: 36331522 PMID: 35255229
Sitagliptin (DPP-4i)	Open-label, RCT	Recent-onset LADA	Preservation of C-peptide (fasting and 2-h postprandial), no difference in HbA1c levels	PIMID: 24432999
Sitagliptin (DPP-4i)	Open-label, RCT	LADA	Stabilized β-cell function (2-h C-peptide), improved HOMA2-B, enhanced insulin secretion (clamp)	PIMID: 33475138
Saxagliptin (DPP-4i) + Vitamin D3	Multicenter pilot RCT (conventional therapy with metformin and/or insulin vs saxagliptin + conventional therapy vs saxagliptin + vitamin D3 + conventional therapy)	LADA	Better preservation of C-peptide (fasting and 2-h); ↓ GAD-Ab titer vs saxagliptin alone or conventional therapy	PIMID: 32043288
Liraglutide (GLP-1 RA)	**ADJUNCT ONE (**phase 3 multicenter RCT, double-blind, placebo-controlled)	Adults with T1D and suboptimal control	↓ HbA1c; ↓ weight; ↓ insulin dose	PIMID: 27506222
Liraglutide (GLP-1 RA)	**ADJUNCT TWO** (phase 3 multicenter RCT, double-blind, placebo-controlled)	Adults with T1D	↓ HbA1c; ↓ weight; ↓ insulin dose	PIMID: 27493132
Semaglutide (GLP-1 RA)	Randomized, double-blind, placebo-controlled crossover trial	Adults with T1D on AID systems	↑ TIR; ↓ insulin dose; ↓ weight	PIMID: 39794615
Semaglutide (GLP-1 RA)	**ADJUST-T1D** (phase 2 RCT, double-blind, placebo-controlled)	Adults with T1D and obesity on AID systems	↑ proportion achieving composite endpoint of TIR >70%, TBR <4% and ≥5% weight loss; greater weight loss vs placebo	PIMID: 40550013
Semaglutide (GLP-1 RA)	**ENDIS** (multicenter RCT, semaglutide or empagliflozin vs placebo)	Adults with T1D	Improvement in endothelial function vs placebo; ↓ hs-CRP (-0.64 ± 6.4, p=0.024) in semaglutide-treated group	PIMID: 37514131

RCT, randomized controlled trial; T1D, type 1 diabetes; LDL-C, low-density lipoprotein cholesterol; HbA1c, glycated hemoglobin; SGLT2i, sodium-glucose cotransporter 2 inhibitors; AID, automated insulin delivery; PLGS, predicted low glucose suspend; TIR, time in range; DPP-4i, dipeptidyl peptidase-4 inhibitor; LADA, latent autoimmune diabetes in adults; HOMA2-B, homeostasis model assessment for β-cell function; GAD-Ab, glutamic acid decarboxylase antibodies; GLP-1 RA, glucagon-like peptide-1 receptor agonist; TBR, time below range; hs-CRP, high sensitivity C-reactive protein.

The main RCTs conducted in subjects with autoimmune diabetes testing anti-diabetic drugs approved for T2D treatment and known to have anti-inflammatory properties were considered. All drugs have been studied as adjunctive therapies to insulin.

Drugs tested and the name of the clinical trial are indicated in bold. ↑ increase; ↓ decrease.

## Challenges and future directions

The progression in the new technologies and the novel immunomodulatory strategies have recently contributed to an overall improvement in the T1D disease management worldwide. However, the disease is progressing toward new endotypes and phenotypes, where autoimmunity is mixed with metabolic dysfunction and ultimately leads to hybrid diabetes conditions, including LADA and DD. Understanding the pathways shared by both forms while identifying mechanisms specifically associated with DD represents an important area of investigation. However, the molecular phenotype of DD remains poorly characterized, thereby limiting its recognition and distinction from other hybrid forms of diabetes, such as LADA. The scientific community is in search for novel disease biomarkers that may help in the diagnosis of such condition given the elevated risk of developing chronic complications that may reduce patients’ life expectancy. If standardized, the multi-omics/genetic approach may help in identifying the “double diabetes risk inflammatory signature”, namely “DRIS”, that can be used to enhance stratification of patients with DD for tailored therapeutic approaches. The repurposing of antidiabetic drugs already approved for obesity and T2D represents an important challenge in the near future to provide patients with hybrid diabetes phenotypes with new treatment opportunities. On the other hand, the discovery of new targets may foster the development of novel strategies and their path toward the clinic, thereby expanding the available therapeutic options for conditions such as DD and LADA. More and larger studies are required in the future to better understand the mechanisms whereby hybrid forms of diabetes develop and whether some therapies may be more beneficial than others.
